# Supplementary material for: Inequities in childhood immunisation coverage associated with socioeconomic, geographic, maternal, child, and place of birth characteristics in Kenya
Source: BMC Infect Dis. 2021 Jun 11;21:553. doi: 10.1186/s12879-021-06271-9 (PMC8192222; doi:10.1186/s12879-021-06271-9)
Supplement: Supplementary file 1 — Additional file 1. [file 12879_2021_6271_MOESM1_ESM.docx]

**Appendix**

**Table A1: Similarities and differences in immunisation coverage estimates for Kenya.** Immunisation coverage in Kenya among children aged 12-23 months based on a nationally representative sample of 3,943 children used in this study and 3,777 children in the Kenya DHS 2014 report^1^. Full immunisation includes 1-dose BCG, 3-dose DTP-HepB-Hib, 3-dose polio, 1-dose measles, and 3-dose pneumococcal vaccines.

| **Vaccine** | **National converge (%)**  (mean and 95% confidence interval – based of this study) | **National converge (%)**  (based of Kenya DHS 2014 report^1^) |
| --- | --- | --- |
| BCG | 96.7 (95.9 - 97.5) | 96.7 |
| DTP-HepB-Hib first dose | 97.4 (96.7 - 98.2) | 97.5 |
| DTP-HepB-Hib second dose | 96.0 (95.0 - 97.0) | 95.8 |
| DTP-HepB-Hib third dose | 90.4 (89.1 - 91.8) | 89.9 |
| Polio first dose | 97.3 (96.6 - 98.0) | 98.0 |
| Polio second dose | 94.6 (93.6 - 95.6) | 96.1 |
| Polio third dose | 82.4 (80.6 - 84.2) | 90.0 |
| Measles | 87.0 (85.7 - 88.3) | 87.1 |
| Pneumococcal first dose | 93.3 (92.0 - 94.6) | 93.7 |
| Pneumococcal second dose | 91.0 (89.5 - 92.5) | 90.8 |
| Pneumococcal third dose | 86.0 (84.5 - 87.6) | 85.1 |
| Full immunisation | 68.2 (66.0 - 70.5) | 74.9 |

^1^ Kenya National Bureau of Statistics. The DHS Program - Kenya: Standard DHS, 2014 [Internet]. 2015. Available: <https://dhsprogram.com/methodology/survey/survey-display-451.cfm>
